# Idiopathic Splenic Artery Pseudoaneurysm Rupture as an Uncommon Cause of Hemorrhagic Shock

**DOI:** 10.1177/2324709615577816

**Published:** 2015-04-13

**Authors:** Richard A. Schatz, Stephen Schabel, Don C. Rockey

**Affiliations:** 1 Department of Medicine, Medical University of South Carolina, Charleston, SC, USA; 2 Department of Radiology, Medical University of South Carolina, Charleston, SC, USA

**Keywords:** hemorrhage, imaging, retroperitoneum, angiography

## Abstract

Splenic artery pseudoaneurysms are infrequently encountered but critical to recognize. Limited literature to date describes associations with pancreatitis, trauma, and rarely peptic ulcer disease. Hemorrhage and abdominal pain are the most common manifestations. There is typically overt gastrointestinal blood loss but bleeding can also extend into the peritoneum, retroperitoneum, adjacent organs, or even a pseudocyst. Most patients with ruptured splenic artery pseudoaneurysms present with hemodynamic instability. Here, we describe a patient recovering from acute illness in the intensive care unit but with otherwise no obvious risk factors or precipitants for visceral pseudoaneurysm. He presented with acute onset altered mental status, nausea, and worsening back and abdominal pain and was found to be in hypovolemic shock. The patient was urgently stabilized until more detailed imaging could be performed, which ultimately revealed the source of blood loss and explained his rapid decompensation. He was successfully treated with arterial coiling and embolization. Thus, we herein emphasize the importance of prompt recognition of hemorrhagic shock and of aggressive hemodynamic stabilization, as well as a focused diagnostic approach to this problem with specific treatment for splenic artery pseudoaneurysm. Finally, we recommend that multidisciplinary management should be the standard approach in all patients with splenic artery pseudoaneurysm.

## Introduction

The sudden development of shock represents a true medical emergency and can be caused by a multitude of different disorders. Major causes of shock include hypovolemic, cardiogenic, and distributive (the latter typically due to an acute and dramatic reduction in systemic vascular resistance). When hypovolemic shock is due to acute blood loss, the source of blood loss is usually obvious. However, the source of blood loss is not always clinically apparent. Here, we present a patient in whom the cause of blood loss was unknown initially but became clear after his symptoms directed specific investigation.

## Case Presentation

A 79-year-old man developed acute-onset altered mental status, nausea, and worsening back and abdominal pain. He had been admitted to the intensive care unit 7 days prior with sepsis from community-acquired pneumonia and was finishing antibiotic treatment with ceftriaxone and azithromycin. His hospital course had been complicated by hypercarbic respiratory failure requiring intubation in the setting of volume overload, but his respiratory status had returned to baseline and he had been extubated 2 days prior.

The past history was remarkable for a chronic obstructive pulmonary disease, gastroesophageal reflux disease, hypertension, hyperlipidemia, coronary artery disease, and abdominal aortic aneurysm. He had an inferior wall myocardial infarction at age 63 years followed by angioplasty with the placement of a single metal stent in the right coronary artery and underwent endovascular repair of the abdominal aortic aneurysm at age 70 years. He smoked 2 packs of cigarettes daily for 50 years but had stopped smoking 10 years ago. He did not use alcohol or illicit drugs. Aside from the antibiotics listed above, his medications included aspirin, simvastatin, furosemide, lisinopril, omeprazole, a budesonide/formoterol inhaler, and albuterol/ipratropium nebulizers.

On physical examination, he was uncomfortable appearing in moderate respiratory distress, pale, and disoriented. His temperature was 36.3°C, blood pressure 86/46 mm Hg, heart rate 110 bpm, respiratory rate 24, and oxygen saturation 93% supplemented on 4 L oxygen via nasal cannula. He had cool, clammy extremities and equal pulses bilaterally. His abdomen was firm, moderately distended, and diffusely tender to palpation with hypoactive bowel sounds. The patient exhibited voluntary guarding but no obvious peritoneal signs. Rectal exam revealed no melena or red blood.

Laboratory data were notable for a hemoglobin of 6.7 g/dL (from 8.8 g/dL 10 hours earlier), hematocrit 22%, white blood count 8700/mm^3^, platelets 270 600/mm^3^, blood urea nitrogen 21 mg/dL, creatinine 0.7 mg/dL, bicarbonate 33 mm/L, international normalized ratio 1.2, and lactate 2.6 mm/L. Serum lipase, aminotransaminases, troponin, and bilirubin levels were normal.

The portable chest radiograph revealed bibasilar atelectasis but a normal cardiac silhouette and mediastinum; an abdominal film showed mildly dilated loops of small bowel but no evidence of obstruction or free air. Electrocardiography showed q waves and nonspecific ST-T wave abnormalities in the inferior leads but this was unchanged from prior tracings. Bedside echocardiography showed hyperdynamic left ventricular function without focal wall motion abnormality. The inferior vena cava measured 1.2 cm in diameter with marked respiratory variation.

**Figure 1.**
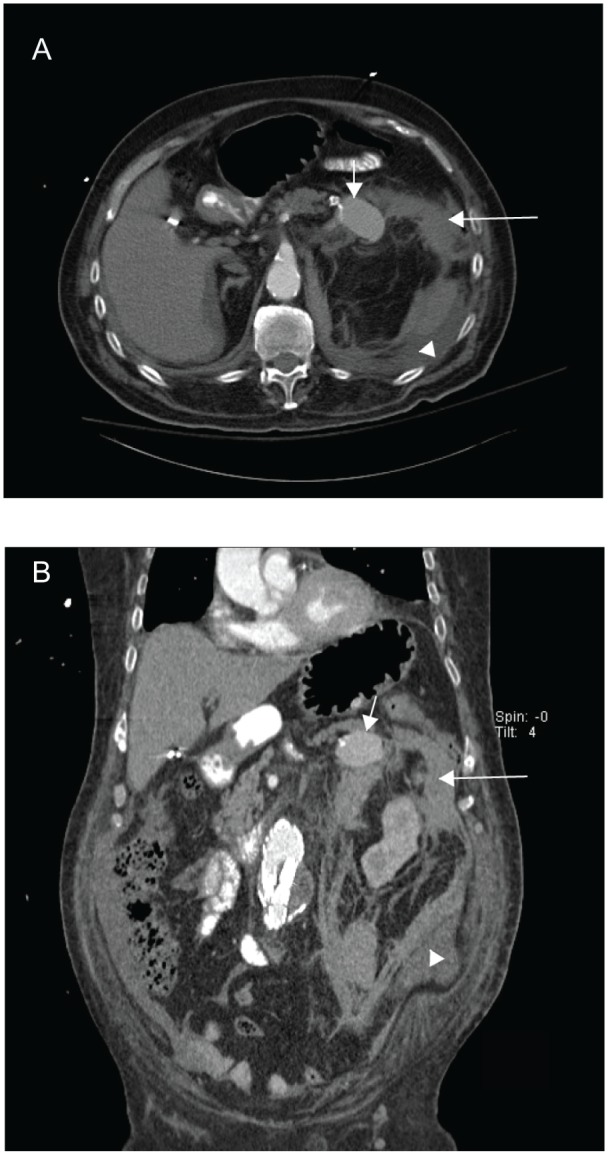
Computed tomography scan of the abdomen and pelvis. (A) An axial view of a contrast-enhanced portal venous phase CT scan demonstrates a 4.5 × 2.6 cm splenic artery pseudoaneurysm (short arrow) with associated retroperitoneal and perinephric fat stranding (long arrow). A dense abdominal free-fluid collection (arrowhead), likely from hemorrhage, is present. (B) A sagittal view of a contrast-enhanced portal venous phase CT scan demonstrates the splenic artery pseudoaneurysm (short arrow), associated retroperitoneal and perinephric fat stranding (long arrow), and the free-fluid collection (arrowhead).

The patient was emergently stabilized by placement of a high-flow non-rebreather oxygen mask and volume resuscitated with 2 L of normal saline and 2 units of packed red blood cells. His vital signs rapidly normalized. Once hemodynamically stable, a computed tomography (CT) scan with contrast of the abdomen and pelvis was obtained and revealed a large left-sided retroperitoneal hematoma with a 4.5 × 2.6 cm contrast-filled structure emanating from the splenic artery consistent with a pseudoaneurysm (Figure 1A and B). Low-density perisplenic and perihepatic free fluid with further fluid extension to the right paracolic gutter and into the pelvis was also noted.

**Figure 2.**
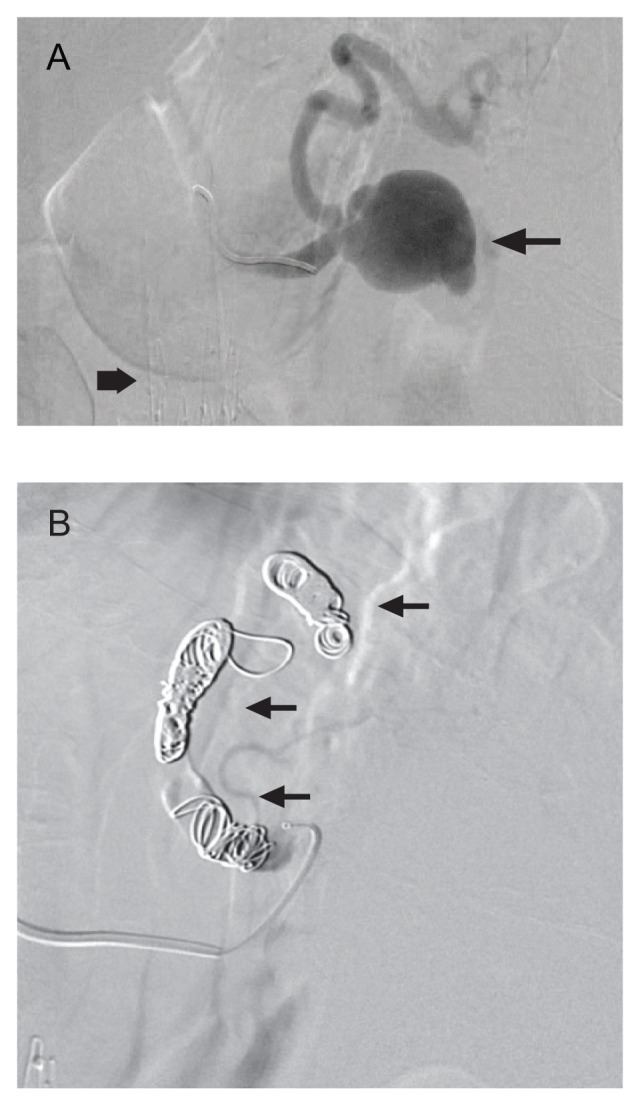
Splenic artery angiography. (A) The digital subtraction image of the splenic artery arteriogram demonstrates extravasation of contrast into a large mid splenic artery pseudoaneurysm (arrow). The angiocatheter is seen in the proximal portion of the splenic artery. Also, an aortic stent graft is also seen (diamond arrow). (B) This digital subtraction image depicts the successfully embolized splenic artery with detachable coils (arrows) throughout the course of the artery. The arteriogram confirms complete stasis of flow within the artery without residual filling of the pseudoaneurysm.

The patient promptly underwent direct catheter angiography, which demonstrated the splenic artery aneurysm and the previously known aortobiiliac stent graft in place (Figure 2A). Subsequently, 14 detachable coils were used to embolize the main splenic artery and occlude the aneurysm (Figure 2B). He required 3 more units of blood throughout the rest of his hospitalization but was discharged home 1 week later without further complication.

## Discussion

Splenic artery pseudoaneurysms are infrequently encountered (<250 cases reported in the literature) but critical to recognize. They are most commonly associated with pancreatitis (acute and chronic), trauma (including iatrogenic causes), and even rarely intra-abdominal processes such as complicated peptic ulcer disease. There are no reported cases of unprovoked, spontaneous splenic artery pseudoaneurysm to date. The clinical presentation varies from incidental finding to acute hemodynamic collapse.^1,2^ Hemorrhage, typically overt with melena and hematochezia from bleeding into the pancreatic duct (known as hemosuccus pancreaticus), and abdominal pain are the most common manifestations.^1^ Ruptured pseudoaneurysms can result in gastrointestinal hemorrhage not only through the pancreatic duct but also into the peritoneum, retroperitoneum, adjacent organs, or even a developing pseudocyst. As in our patient, the majority of patients with active bleeding are hemodynamically unstable at the time of presentation. Splenic artery pseudoaneurysms carry a much higher risk of rupture than true splenic artery aneurysms since they lack at least one component of the traditional arterial wall (adventitia, media, intima). Prompt detection and intervention is critical as these carry up to a 90% mortality rate if untreated.^3^

At presentation, the patient’s sudden development of tachycardia, hypotension, and altered mental status is consistent with shock. Shock, broadly characterized into 3 major categories (Table 1), is a physiologic state characterized by systemic reduction in tissue perfusion resulting in poor tissue oxygen delivery, which can progress to end-organ dysfunction. Whereas distributive (septic, neurogenic, or anaphylactic) shock manifests as a consequence of decreased systemic vascular resistance, cardiogenic shock is due to reduced cardiac output due to pump failure or decreased stroke volume, and hypovolemic shock from a global reduction in cardiac preload. Recognizing the presentation and specific type of shock is essential to clarifying the etiology of his acute picture. Most notable is the substantial drop in hemoglobin and hematocrit, which suggests hypovolemic shock secondary to acute blood loss. Stabilizing the patient with suspected hypovolemic shock from active blood loss is the most important initial priority. Ensuring a patent and protected airway and establishing large bore intravenous access are paramount.

**Table 1.** Categories of Shock.

Category	Physiology	Common Causes	Basic Treatment
Hypovolemic	↓ Cardiac preload and cardiac output	• Hemorrhage	First line: Volume resuscitation
		• Fluid losses (GI, burns)	Second line: Vasopressors
		Dehydration	
Cardiogenic	↓ Stroke volume and cardiac output	Intracardiac	• Treat underlying cause
		• Myocardial infarction	• Aggressive volume management (ie, bolus vs dieresis depending on etiology)
		• Arrhythmia (ie, atrial fibrillation with rapid ventricular response, unstable ventricular tachycardia, ventricular fibrillation)	• Vasopressors ± inotropes
		• Aortic stenosis	
		• Mitral regurgitation	
		Extracardiac	
		• Volume overload	
		• Tension pneumothorax	
		• Massive pulmonary embolus	
		• Cardiac tamponade	
Distributive (septic, anaphylactic, neurogenic)	↓ Systemic vascular resistance from ↑ vasodilatation	Severe inflammatory response with massive cytokine release (ie, infection, pancreatitis, burns), allergic reaction, spinal injury/anesthesia	First line: Volume resuscitation
			Second line: Vasopressors
			±: Antibiotics, steroids/epinephrine

The degree of intravascular volume depletion, classified in stages I to IV of hypovolemic shock, is important to understand as this directs resuscitative measures. The body can initially compensate up to ~15% reduction in blood volume (stage I) via peripheral arterial vasoconstriction. However, compensatory mechanisms become overwhelmed with the loss of 15% to 30% of circulating blood volume (stage II). Acute loss of 30% to 45% blood volume (stage III) best characterizes this patient’s presentation with his low systolic blood pressure (<100 mm Hg), marked tachycardia, tachypnea, altered mental status, and cool extremities. Prompt recognition can prevent progression to profound hypotension and end-organ collapse (stage IV).

This patient’s situation was remarkable due to his rapid decompensation without overt blood loss. While the differential diagnosis for hypovolemic shock without external blood loss is wide, it can be quickly focused. His abnormal abdominal physical examination and history of known vascular disease initially raised the possibility intra-abdominal sepsis due to many different causes (ie, abscess, hepatobiliary disease, perforation of a viscous), acute mesenteric ischemia, an acute vascular disorder (involving the spleen, pancreas, or kidneys), hemorrhagic pancreatitis, or colitis. Although the absence of melena, hematochezia, or hematemesis argued against hemorrhage within the gastrointestinal lumen, intra-abdominal blood loss required a thorough evaluation.

The patient’s prior history of repaired abdominal aortic aneurysm was concerning for aneurysmal hemorrhage. The triad of abdominal pain, shock, and a pulsatile abdominal mass essentially confirms a ruptured abdominal aortic aneurysm, a surgical emergency. Indeed, the patient complained of worsening abdominal and back pain and met shock criteria but a thorough abdominal examination did not reveal an underlying pulsatile mass. Still, rapidly increasing abdominal distention in the setting of hemodynamic instability was concerning for developing abdominal compartment syndrome, also a surgical emergency. Type 1 aortic dissection can also present similarly in an elderly man with hypertension and atherosclerotic disease. Likewise, the prior history of significant coronary artery disease warranted evaluation for acute myocardial infarction causing cardiogenic shock. Although unlikely in this particular case, patients with large declines in hematocrit that cannot be readily explained may have more chronically occult or obscure gastrointestinal bleeding as the gastrointestinal tract is large and blood can be hidden within it or unable to be visualized with standard endoscopic procedures.^4^ In the absence of clear gastrointestinal tract bleeding, several other disorders including large volume fluid shifts, occult sepsis, severe hemolytic anemia, and large hematomas should also be considered.^5^

If visceral aneurysm and pseudoaneurysm are suspected, the distinction between these 2 entities is important since this guides further clinical decision making. Splenic artery aneurysms differ from pseudoaneurysms in etiology, natural history, outcome, and management. The distinction can often be made radiographically as true aneurysms typically show evidence of atherosclerosis and calcification and pseudoaneurysms often hold a multilobular appearance. The splenic artery is the third most common location for true intra-abdominal aneurysm and the vast majority are discovered incidentally. Their incidence has increased with more widespread use of advanced imaging.^6^ Similar to abdominal aortic aneurysm, splenic artery aneurysm has hypothesized associations with atherosclerosis and hypertension, but can also be encountered with portal hypertension and cirrhosis, previous liver transplant, and pregnancy.^7,8^ While the risk of rupture is fairly low (2% to 3%), ruptured aneurysms have potentially catastrophic consequences.^9^ Current practice favors treating asymptomatic aneurysms greater than 2 cm.^7,8^

This case also highlights the utility of CT absorption density in determining the nature of abdominal fluid collections. The density of the blood within the abdominal aorta measures 250 Hounsfield units, typical of arterial blood in the portal venous phase of enhancement. The pseudoaneurysm contents measured 140 Hounsfield units, typical of portal venous phase enhanced blood mixed with arterial blood. The absence of gas suggests that the lesion is not a gastrointestinal fistula, and the lack of very high-density oral contrast suggests that the lesion does not communicate with the gastrointestinal tract. Of note, the perisplenic ascites measured 14 Hounsfield units, most compatible with extracellular fluid that contains a mix of cellular and/or blood proteins.

Standard evaluation and treatment for splenic pseudoaneurysm is angiographic-directed embolization (with either coils, inert particles, or gel-foam),^10,11^ which the patient underwent after the initial CT scan. While CT angiography and magnetic resonance angiography may offer better resolution, these methods do not allow concurrent therapeutic intervention. However, these advanced imaging methods can only be performed in patients with adequate renal function since they require the administration of intravenous contrast. In the past, splenic artery ligation and splenectomy with or without partial pancreatectomy were most commonly performed but novel, less invasive methods are now favored with better outcomes and less morbidity.^12^ CT-guided thrombin injection can be used if patients are not candidates for endovascular treatment.^13^ There is little correlation between size and risk of rupture; hence, once diagnosed, prompt intervention should be sought.^1^

In summary, hemorrhagic shock caused by splenic artery pseudoaneurysm without sentinel gastrointestinal bleeding is relatively uncommon but critical to accurately diagnose and manage. Although the initial diagnosis in this patient was unclear, this patient’s presentation highlights the necessity of prompt recognition of hemorrhagic shock, the importance of aggressive stabilization, and, subsequently, a focused diagnostic approach with specific treatment. In this particular disorder, multidisciplinary involvement (with interventional radiology and surgical consultation) should be sought early.

## References

[1] Tessier DJ Stone WM Fowl RJ Clinical features and management of splenic artery pseudoaneurysm: case series and cumulative review of literature. J Vasc Surg. 2003;38:969-974. 14603202 10.1016/s0741-5214(03)00710-9

[2] Nicaise N Golzarian J van Gansbeke D Cremer M Struyven J Deviere J Rupture of pseudoaneurysm: a cause of delayed hemorrhage after endoscopic cystoenterostomy; angiographic diagnosis and treatment. Gastrointest Endosc. 1998;47:186-189. 9512288 10.1016/s0016-5107(98)70356-6

[3] Huang IH Zuckerman DA Matthews JB Occlusion of a giant splenic artery pseudoaneurysm with percutaneous thrombin-collagen injection. J Vasc Surg. 2004;40:574-577. 15337894 10.1016/j.jvs.2004.06.020

[4] Rockey DC Occult gastrointestinal bleeding. N Engl J Med. 1999;341:38-46. 10387941 10.1056/NEJM199907013410107

[5] Ong B Rockey DC The syndrome of a large drop in hematocrit in hospitalized patients: clinical features and gastrointestinal bleeding outcomes. J Investig Med. 2014;62:963-967. 10.1097/JIM.0000000000000109 25203151

[6] Røkke O Søndenaa K Amundsen S Bjerke-Larssen T Jensen D The diagnosis and management of splanchnic artery aneurysms. Scand J Gastroenterol. 1996;31:737-743. 8858739 10.3109/00365529609010344

[7] Agrawal GA Johnson PT Fishman EK Splenic artery aneurysms and pseudoaneurysms: clinical distinctions and CT appearances. AJR Am J Roentgenol. 2007;188:992-999. 17377035 10.2214/AJR.06.0794

[8] Abbas MA Stone WM Fowl RJ Splenic artery aneurysms: two decades experience at Mayo Clinic. Ann Vasc Surg. 2002;16:442-449. 12089631 10.1007/s10016-001-0207-4

[9] Mattar SG Lumsden AB The management of splenic artery aneurysms: experience with 23 cases. Am J Surg. 1995;169:580-584. 7771620 10.1016/s0002-9610(99)80225-6

[10] Arepally A Dagli M Hofmann LV Kim HS Cooper M Klein A Treatment of splenic artery aneurysm with use of a stent-graft. J Vasc Interv Radiol. 2002;13:631-633. 12050305 10.1016/s1051-0443(07)61659-5

[11] Guillon R Garcier JM Abergel A Management of splenic artery aneurysms and false aneurysms with endovascular treatment in 12 patients. Cardiovasc Intervent Radiol. 2003;26:256-260. 14562974 10.1007/s00270-003-1948-y

[12] Dave SP Reis ED Hossain A Taub PJ Kerstein MD Hollier LH Splenic artery aneurysm in the 1990s. Ann Vasc Surg. 2000;14:223-229. 10796953 10.1007/s100169910039

[13] Krueger K Zaehringer M Lackner K Percutaneous treatment of a splenic artery pseudoaneurysm by thrombin injection. J Vasc Interv Radiol. 2005;16:1023-1025. 16002512 10.1097/01.RVI.0000162167.54455.C0

